# Improved method for enumerating sulfate-reducing bacteria using optical density

**DOI:** 10.1016/j.mex.2015.04.006

**Published:** 2015-04-24

**Authors:** L.A. Bernardez, L.R.P. de Andrade Lima

**Affiliations:** a Graduate Program of Industrial Engineering, Federal University of Bahia, Brazil; b Department of Materials Science and Technology, Federal University of Bahia, C.P. 6974, Salvador, BA 41810-971, Brazil

**Keywords:** Reducing interference in the analysis of the sulfate-reducing bacteria solution content by optical density using acidified solutions, Sulfate-reducing bacteria, Souring, Iron sulfide, Optical density

## Abstract

The photometric determination of bacterial concentration can be affected by secondary scattering and other interferences. The conventional growth medium for sulfate-reducing bacteria (SRB) has iron that precipitates as iron sulfides, a dark precipitate which is useful to indicate bacterial activity. However, iron hydroxides also precipitate at high pH values and the presence of these precipitates interferes considerably in the optical density of the solution affecting estimates of the cell population thus seriously limiting the use of the conventional method. In this method a modification of the current method improves the measurement of the optical density of a solution with SRB cells.

• The method consists of an acidification with hydrochloric acid of a sample of a mixed culture of SRB enriched from the produced water from oil fields to pH below 2.
• The results show that the relationship between the bacterial dry mass and absorbance is exponential in the observed range. It was observed a large slope in the linearized fit equation, and the acidified solution does not change the integrity of the SRB cells after the treatment.
• The results of the kinetic experiments, including the bacterial growth time evolution, demonstrate the applicability of the method.

## Graphical abstract

**Figure:**
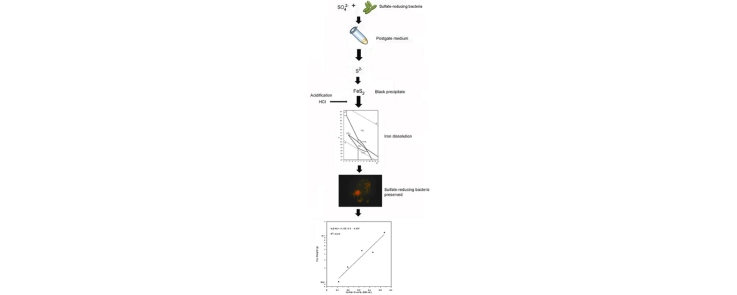

## Methods details

### Microbial culture and medium

A mixed culture of sulfate reducing bacteria was enriched from the produced water from the oil fields of the Recôncavo Baiano basin, Brazil and used as an inoculum. This enrichment is dominated by bacteria of the species *Desulfovibrio vulgaris* [1], [2], [3].

### Medium

Inside the anaerobic chamber (Bactron VI, Shellab, Sheldon Manufacturing, Incorporation using a mixed gas of 5% hydrogen, 5% carbon dioxide, and 90% nitrogen) at 38 °C a volume of 100 μL of mix culture of SRB was inoculated and enriched in culture standard medium. The Postgate standard medium was used for the growth of the SRB; for the maintenance a sulfate poor Postgate medium, agar was used and it is referred as the modified Postgate medium. Sodium lactate was used as a carbon source. Blackening of the medium due to the formation of iron sulfides was indicative of positive growth.

The Postgate standard medium contained per L: agar, 2.0 g (Difico); KH_2_PO_4_, 0.5 g (Merk); NH_4_Cl, 1.0 g (J.T. Backer); Na_2_SO_4_, 1.0 g (Merk); CaCl_2_, 1.0 g (Vetec); MgCl_2_·6H_2_O, 1.83 g (Merk); yeast extract, 1.0 g (Merk); ascorbic acid, 0.1 g (Merk); sodium thioglycolate, 0.013 g (Vetec); sodium citrate, 6.38 g (Synth); sodium lactate 1.75 mL; NaCl (Quemis) 3.5%, resazurin, 2.0 mL (Vetec) 0.025% P/V, FeSO_4_·7H_2_O, 0.5 g (Merk). All the components were dissolved in distilled water and the pH was adjusted to 7.5–8.0 using 5 M HCl. After this, the solution was homogenized by agitation and later sterilized at 121 °C for 30 min.

### Removal of iron precipitates by acidification

Aliquots of 2.8 mL were transferred to an Erlenmeyer flask containing 250 mL of Postgate medium. The Erlenmeyer was incubated at 38 °C in an anaerobic environment (Bactron Anaerobic/Environmental Chamber—Shel Lab) for 120 h for optimal growth. Aliquots of 45 mL were taken and 2 mL of hydrochloric acid was added to the sample. After 15 min, the culture was centrifuged at 10 °C for 20 min at 11,000 RPM (the relative centrifuge force was 17,316 × *g*). The supernatant was discarded and the biomass was washed with 10 mL of distilled water. The suspension was centrifuged at 10 °C for 10 min at 10,000 RPM (the relative centrifuge force was 14310 × *g*). The supernatant was again discarded and the biomass was resuspended using 10 mL of distilled water to produce a start bacterial suspension.

From the start bacterial suspension a 1:10 dilution was made containing 1 mL of the start bacterial suspension and 9 mL of distilled water. From the 1:10 dilution, subsequent dilutions were made and were used in the construction of the curve. The dilutions were the following (in duplicate): 1:5, 1:10, 1:15, 1:20, 1:25. After this, the optical density of the samples and respective dry weights were analyzed.

### Dry weight measurement

The filter membranes of cellulose acetate with pore size of 0.22 μm were dried in an oven in an empty aluminum weighing pan. After this, they were weighed and stored in a desiccator lined with anhydrous CaSO_4_ for 24 h. Then 5 mL of the culture was poured into the holding reservoir fitted on the filter membrane. A vacuum was applied to pull the liquid through the membrane. The reservoir was rinsed with a few milliliter of water and any paste adhering to the glassware was scraped off. The wet weight of the culture was measured immediately after all the water has been pulled through. The cell paste was dried in an oven set at 105 °C. The weight of the pan/filter plus the cell paste was measured periodically until there was no further decrease in the dry weight. It took 24 h to dry the sample completely. The difference in the weight was calculated, and the dry weight was expressed in g/L.

### Optical density

The samples were diluted to appropriate concentrations as needed and the absorbance of the sample was measured with a spectrophotometer at 600 nm. A calibration curve to relate the absorbance with cell dry weight was then generated. As a rule of thumb, an optical density of one unit corresponds to approximately 1.0 g/L of dry cell. This is also commonly referred to as the turbidity measurement.

### Acridine orange method

SRB were grown in a modified Postgate medium and the cells were harvested in the late exponential phase. Aliquots of 1.5 mL were taken from the cell culture and added to a clean test tube. The cells were centrifuged at 10,000 RPM (the relative centrifuge force was 14,310 × *g*) for 10 min. The cells were washed twice in buffer PBS and resuspended in 1.0 mL of acridine orange stock solution (0.1 g of AO in 100 mL of sterilized distilled water). Then the sample was covered with aluminum foil and left to stand for 30 min in the dark. A 10 μL sample was spread on a clean microscope glass slide and immediately analyzed for fluorescence in a microscope (Olympus BX51).

### Bacterial growth evaluation

The kinetics were investigated in sealed 250 mL glass bottles in which an equal amount of 200 mL of the previously autoclaved medium, without agar, and 50 mL of inoculum were added and put inside the anaerobic chamber. Then the bottles were continuously mixed in a mechanical shaker at 120 RPM at 38 °C. At certain time intervals, one bottle was selected, aliquots withdrawn and used for the chemical and biological analysis. The amount of Na_2_SO_4_ and FeSO_4_·7H_2_O of the modified Postgate medium were proportionately modified in the culture medium to reach the concentrations of 1790 mg/L; the amount of the other components of the culture medium was the same.

**Fig. 1:**
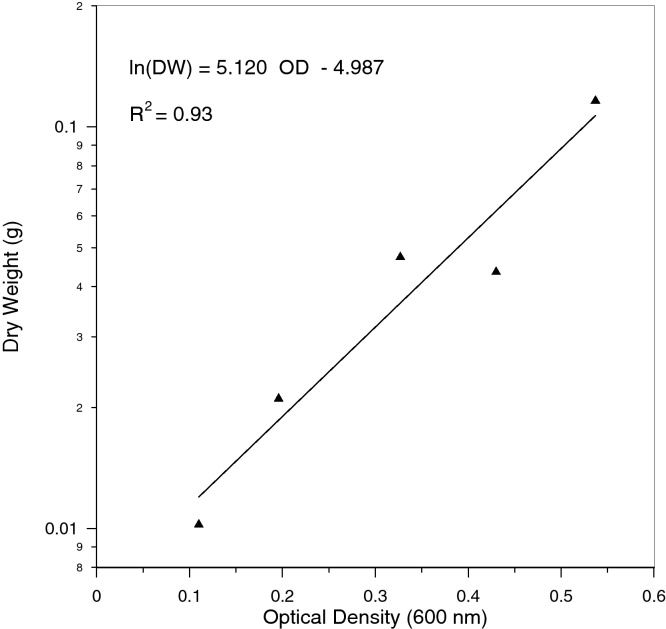
Cell concentration (DW) as a function of optical density (absorbance at 600 nm).

During the kinetic experiment samples were withdrawn at suitable time intervals and then after acidification the biomass concentrations are evaluated using the calibration curve in Fig. 1. The sulfate and sulfide solution content was measured by turbidimetric methods. Barium sulfate was added to the samples to precipitate the sulfate ions as barium sulfate and copper sulfate was added to precipitate the sulfide ions as copper sulfide. For the pH measurements a pH meter calibrated using buffer solutions of pHs of 4 and 7 was used. The redox potentials were measured using an ORP electrode with an internal Ag/AgCl reference electrode [1], [2].

## Method validation

After aliquots of the culture mix were transferred to the Erlenmeyer flask containing 250 mL of Postgate medium and incubated at 38 °C in anaerobic environment for 120 h for optimal growth, a distinct black precipitate became visible after some time. This precipitate could not be readily removed by filtration or centrifugation. The addition of hydrochloride acid (HCl) to the mixture until pH 2 resulted in a rapid reaction, leading to a color change from a dark brown color of FeS, FeS_2_ and FeOOH to a clear solution of Fe^2+^.

Fig. 1 shows the relationship between the cell concentration expressed as dry biomass and absorbance, reading at 600 nm of SRB cells growing in Postgate standard medium with the addition of HCl to a pH value below 2. In the presence of the acid the experimental data fitted the predicted curve well with a correlation coefficient equal to 0.98. The results with no addition of acid, not shown here, fitted poorly which confirms that the acidification of the medium reduces the iron precipitation and the interference thereof considerably.

**Fig. 2:**
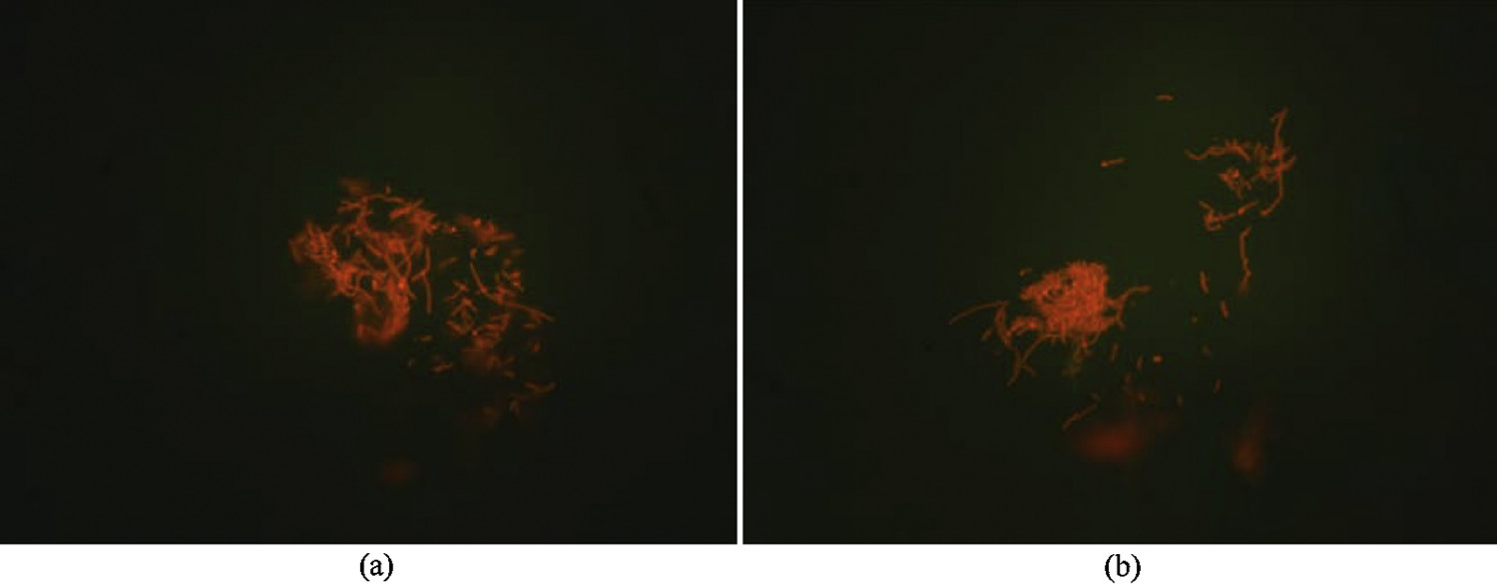
Identification of SRB cells by fluorescence microscopy and acridine orange staining. (a) Before the acidification. (b) After the acidification.

Fig. 2a and b shows microscopic images of the SRB cells. Fig. 2a shows the SRB cells before acidification and Fig. 2b shows the SRB cells after acidification. After acidification the quality of the image is enhanced while the integrity of the cells seems to be preserved.

**Fig. 3:**
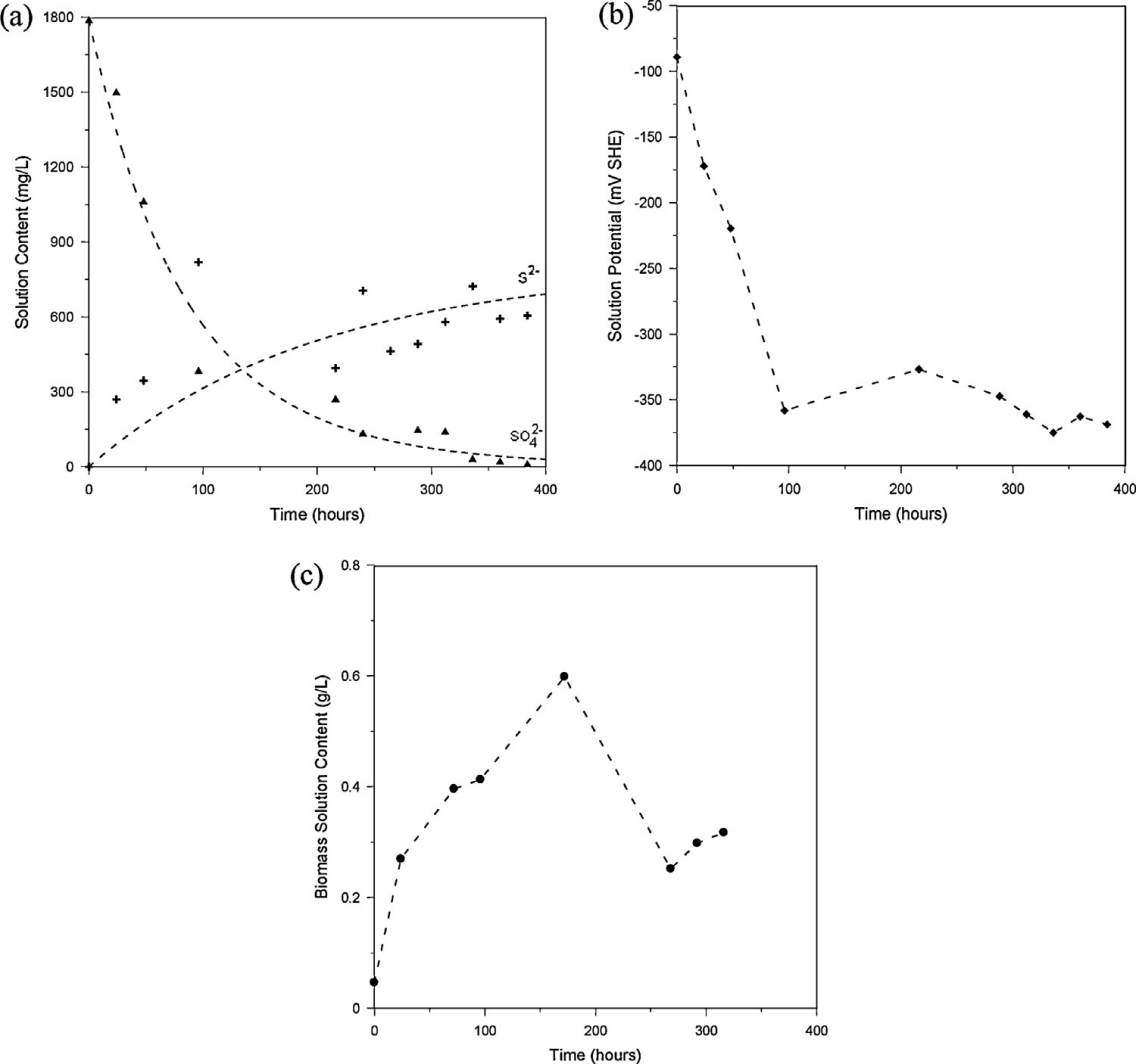
Batch kinetic tests for bioconversion of sulfate using SRB: (a) sulfate and sulfide solution content time evolution, (b) solution oxidation–reduction potential time evolution. (d) Biomass solution content time evolution.

The bioprocess kinetics were determined in a batch reactor with an initial sulfate concentration of 1790 mg/L. Fig. 3a shows the profiles of residual sulfate (filled triangle symbols) and produced sulfide (cross symbols) concentrations as a function of time. In 360 h (15 days) the sulfate content decreased from 1790 to 22.2 mg/L (a conversion of about 98.8%) and the sulfide concentration increased to 592.6 mg/L. Fig. 3b shows the redox potential measurement (filled diamond symbols), which was used to indicate the bacterial activity due to the reducer character of the SRB. During this period, a gradual decrease in the solution redox potential was observed. Fig. 3c shows the biomass solution content (filled circle symbols) estimated using the methods presented in this study. One remarks the enhancement of the microbial solution content, which is correlated with the redox potential reduction and the sulfide formation.

## Additional information

### Background

Sulfate-reducing bacteria are comprised of a group of morphologically diverse and anaerobic organisms that obtain their energy by dissimilatory reduction of sulfate to sulfide [1], [2]. They are important for a number of economic and ecological reasons. In relation to the oil industry they inhabit the water base of oil bearing strata, injection waters used in secondary oil recovery and drilling muds used in exploration for oils. They cause corrosion of pumping machinery and storage tanks, pollute oil products by introducing reduced sulfur into oil and its associated gas, produce H_2_S gas result in souring of oil and gas, and rock-pore blockage by bacterial cells and precipitates of iron sulfides [3]. Any ecological study of SRB requires their detection and enumeration [4], [5], [6].

The cell density can be quantified in two basic ways: as the number of viable/dead cells per mL and as grams of dry or wet weight per liter of sample. The number of cells can be counted either by successively diluting the original sample and plating on a Petri dish, with the help of a microscope and a counting chamber, or with an automated cell counter.

In practical fermentation or during kinetic studies these methods are not desirable as all of the above methods either require the availability of equipment or substantial investment of time. For example, the standard plate count can detect viable cells among other particulate matter. However, the method requires elaborate preparation and it takes 24–48 h for the cells to be incubated and counted; the cost of Petri dishes and media can also be prohibitive. Consequently, the direct plate count is useless in feedback control of a fermentation process and it is mainly used industrially to countercheck other measurements, especially optical density. In fact the most frequently used method simply monitors the optical density of the sample. The absorbance of the sample measured in a spectrophotometer is correlated to either the dry weight or the number of cells per volume.

The determination of biomass or cell numbers in microbial cultures can be based on viable counts, biochemical determinations, microscopic counting or usually on turbidity measurements. The photometric determination of bacterial concentration, which uses the Lambert–Beer law of absorption (optical density $\left(OD\right)=\log I_{0}/I=kc$), can have proportionality only at low concentration ranges due to secondary scattering and other interferences.

Biomass concentration is one of the most critically needed measurements in fermentation studies and kinetic experiments. It is also one of the most difficult and sometimes unreliable ones. For example, the optical density measurement fails completely if the sample contains other insoluble particulate matter, which is often the case in a practical fermentation reactor and in an oil-field brine sample. Similarly, the optical density measurement only has limited usefulness if the sample is not clear.

**Fig. 4:**
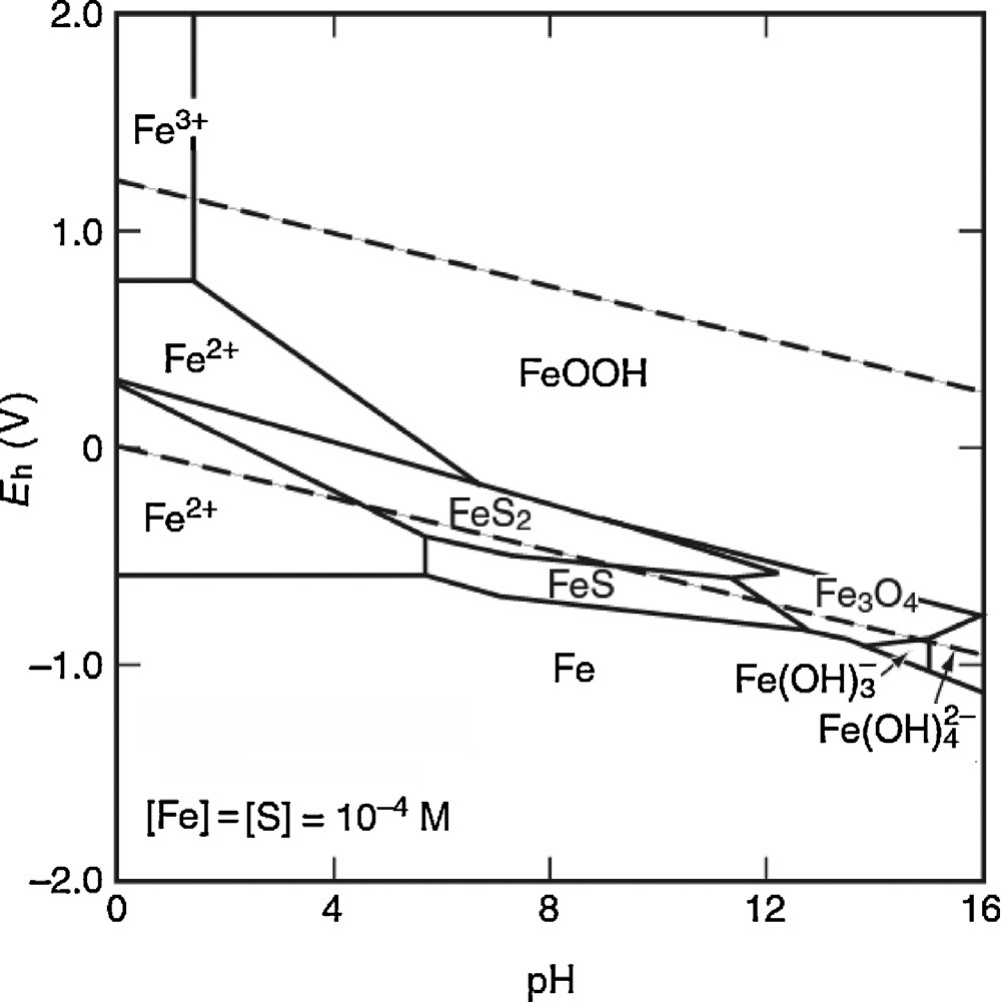
The Eh–pH diagram for Fe-S-H_2_O system at 25 °C (adapted from [8]).

In studies involving sulfate reducing bacteria (SRB) isolation, growing and numeration from oil-fields samples, Postgate B medium is widely used [7]. This medium is a general purpose medium for detecting and culturing several SRB such as *Desulfovibrio* and *Desulfotomaculum*. However, the photometric measurements of the concentration of cells in this medium is affected by the amount of dark iron sulfides precipitate (FeS and FeS_2_) present in the Postgate medium, which causes the low reproducibility of the standard curves (see Fig. 4).

An enumeration technique based on microscope images is also affected by the precipitate leading to uncertainty of the results. Centrifugation of the sample at progressive speeds to remove the precipitate is a common technique employed to circumvent this possible error but the use of this technique is time and reagent consuming [9]. This paper presents a way to overcome this problem. The method described here involves a direct procedure suitable for enumerating SRB in mixed culture from an oil-field sample. The procedure is quick and simple, efficient with respect to time, reagent and equipment, yet it demonstrates high sensitivity.
